# Data for erring patterns in manual delineation of PET-imaged lung lesions

**DOI:** 10.1016/j.dib.2019.104846

**Published:** 2019-11-20

**Authors:** Fei Yang, Lori Young, Yidong Yang

**Affiliations:** a Department of Radiation Oncology, University of Miami, Miami, FL, USA; b Department of Radiation Oncology, University of Washington, Seattle, WA, USA; c The First Affiliated Hospital of University of Science and Technology of China, Hefei, PR China

**Keywords:** Quantitative PET, Target delineation, Segmentation evaluation, Lung cancer

## Abstract

The data presented in this article characterizes the erring patterns intrinsic to manual contouring of PET positive tumor targets in the lung from twelve quantitative agreement measuring metrics, with categories related respectively to spatial overlap, pair counting, information theory, distance, and volume. The data holds the potential for the formation of new hypotheses towards improving the accuracy and precision of manual delineation of PET positive lung targets for radiation therapy.

**Table:** Specifications Table

Subject area	*Radiation Oncology*
More specific subject area	*Target Delineation*
Type of data	*Table*
How data was acquired	*Data was obtained by comparing manual contouring results against ground truth*
Data format	*Raw, analyzed, and descriptive*
Experimental factors	*PET images were generated based on Monte Carlo simulations and target contours were provided by physician raters*
Experimental features	*Segmentation accuracy of manual contouring assessed by 12 quantitative agreement metrics*
Data source location	*Department of Radiation Oncology, University of Miami, Miami, FL, USA*
Data accessibility	*Data is included within the article*
Related research article	*F Yang, L Young, Y Yang. Quantitative imaging: Erring patterns in manual delineation of PET-imaged lung lesions. Radiotherapy and Oncology (in press)*

**Table:** 

**Value of the Data** • The data will be informative to clinicians towards improving the accuracy and precision of the definition of PET positive tumor target in the lung. • The data allows to understand and interpret the erring patterns inherent to manual contouring of PET-imaged lung lesion. • The data illustrates that different accuracy measuring metrics evaluate the goodness of the segmentation from different perspectives and evidences the dependency of segmentation evaluation on the choice of accuracy measures. • The data allows the chance to gain understanding of the inter/intra-rater variabilities in delineating radiotherapy target volumes on PET for lung cancer at a larger cohort level. • The data presented significantly extends our previous report [1], and thus furnishing more comprehensive and comprehensible views upon the accuracy of manual delineation of PET positive lung targets.

## 1 Data

The data presented in this article (Table 1, Table 2, Table 3, Table 4, Table 5, Table 6, Table 7, Table 8, Table 9, Table 10, Table 11, Table 12) characterizes the accuracy of manual delineation of PET positive tumor target in the lung by ten physician raters within the context of complete known ground truth from twelve well-established agreement measuring metrics, with categories related respectively to spatial overlap, pair counting, information theory, distance, and volume. Spatial overlap was measured with using Dice coefficient (DICE), false negative dice (FND), false positive dice (FPD), and global consistency error (GCOERR). Pair counting based metrics measure the agreement by counting corrected segmented pairs of voxels and consist of Rand index (RNDIND) and adjusted Rand index (ADJRIND). Metrics based on information theory quantify the statistical dependency and include normalized mutual information (NMUTINF) and normalized variation of information (NVARINFO). Spatial distance was assessed via three metrics including symmetric mean absolute surface distance (SMASD), average Hausdorff distance (AHDST), and Mahalanobis Distance (MDST). Absolute volumetric difference (AVD) was used to evaluate the extent to which manual contour deviates from ground truth in volumes. The data illustrates the various underlying aspects of the behavior patterns of manual contouring of PET-imaged lung lesion and allows the potential for the formation of new hypotheses towards improving the accuracy and precision of manual delineation of PET positive lung targets.

**Table 1:** Dice coefficient (DICE) between the manual contour of individual raters and the ground truth for each of the simulated PET lesion. DICE evaluates to 1 for ideal segmentation.

Lesion	Rater									
	R-1	R-2	R-3	R-4	R-5	R-6	R-7	R-8	R-9	R-10
L-1	0.9389	0.8822	0.9415	0.8797	0.9040	0.7565	0.9317	0.9200	0.9161	0.9178
L-2	0.9340	0.8872	0.9140	0.8404	0.8929	0.8289	0.8926	0.9150	0.9219	0.9128
L-3	0.9287	0.8957	0.8316	0.9010	0.8833	0.9068	0.8475	0.8939	0.9340	0.9330
L-4	0.8883	0.8515	0.8779	0.8847	0.8818	0.8340	0.8458	0.8779	0.8989	0.9125
L-5	0.9176	0.8431	0.9216	0.8304	0.8879	0.8801	0.8208	0.8991	0.8916	0.9167
L-6	0.9386	0.9341	0.9106	0.8654	0.8561	0.8437	0.9066	0.9429	0.9285	0.9449
L-7	0.7841	0.7530	0.7506	0.7034	0.7322	0.7380	0.7061	0.8184	0.7629	0.7328
L-8	0.9027	0.8141	0.9237	0.8897	0.8524	0.8788	0.8503	0.8624	0.8675	0.9081
L-9	0.9124	0.8516	0.9391	0.8962	0.8944	0.8796	0.8898	0.9230	0.9115	0.9050
L-10	0.8797	0.9059	0.9561	0.9086	0.8777	0.9259	0.8491	0.9059	0.8955	0.9045
L-11	0.8895	0.8341	0.8993	0.8508	0.8106	0.8000	0.8037	0.9005	0.8889	0.8890
L-12	0.8746	0.8325	0.8308	0.8629	0.8138	0.7182	0.8428	0.7668	0.7806	0.8210
L-13	0.8476	0.9010	0.9097	0.8818	0.8648	0.8692	0.7299	0.9256	0.9116	0.9044
L-14	0.8424	0.7617	0.8883	0.8752	0.8327	0.8411	0.8171	0.8782	0.8281	0.8490
L-15	0.8524	0.8608	0.9217	0.8709	0.8748	0.7972	0.8430	0.9295	0.9376	0.8297
L-16	0.8277	0.8594	0.8565	0.8578	0.8327	0.8033	0.7497	0.8934	0.8675	0.9312
L-17	0.9269	0.9045	0.9283	0.9236	0.9169	0.9244	0.9055	0.9377	0.9554	0.9083
L-18	0.8698	0.8426	0.8666	0.8526	0.8629	0.8198	0.8504	0.9000	0.9245	0.8998
L-19	0.8839	0.8936	0.9040	0.9162	0.9179	0.9128	0.8560	0.9378	0.9433	0.9072
L-20	0.8115	0.7685	0.8722	0.8881	0.8581	0.8712	0.7369	0.9335	0.9303	0.8620
L-21	0.8169	0.7367	0.8774	0.9002	0.8776	0.8618	0.7579	0.9131	0.8997	0.8407
L-22	0.8728	0.7455	0.8716	0.8859	0.8779	0.8498	0.8503	0.9203	0.9236	0.8314
L-23	0.8333	0.7901	0.8310	0.8400	0.8593	0.8584	0.8506	0.8741	0.8602	0.8579
L-24	0.7218	0.6813	0.7763	0.8003	0.7733	0.8093	0.7191	0.7881	0.8337	0.7499
L-25	0.8214	0.7303	0.8557	0.8929	0.8505	0.8818	0.8540	0.8988	0.9059	0.8532
L-26	0.7982	0.7538	0.8597	0.8778	0.8901	0.8527	0.8348	0.9014	0.8997	0.8367

**Table 2:** False negative Dice (FND) between the manual contour of individual raters and the ground truth for each of the simulated PET lesion. FND evaluates to 0 for ideal segmentation.

Lesion	Rater									
	R-1	R-2	R-3	R-4	R-5	R-6	R-7	R-8	R-9	R-10
L-1	0.0310	0.0105	0.0090	0.0014	0.0051	0.0000	0.0143	0.0359	0.0083	0.0025
L-2	0.0406	0.0386	0.0048	0.0040	0.0165	0.0015	0.0077	0.0215	0.0273	0.0119
L-3	0.0524	0.0080	0.0014	0.0024	0.0015	0.0024	0.0000	0.0442	0.0041	0.0066
L-4	0.0115	0.0012	0.0037	0.0076	0.0037	0.0182	0.0085	0.0037	0.0038	0.0158
L-5	0.0243	0.0079	0.0067	0.0008	0.0102	0.0073	0.0034	0.0745	0.0150	0.0076
L-6	0.0455	0.0452	0.0031	0.0007	0.0000	0.0029	0.0000	0.0253	0.0131	0.0494
L-7	0.0000	0.0000	0.0000	0.0000	0.0000	0.0000	0.0000	0.0053	0.0000	0.0000
L-8	0.0969	0.2877	0.0724	0.0412	0.1967	0.2032	0.0265	0.2215	0.2316	0.0681
L-9	0.0415	0.0434	0.0129	0.0000	0.0012	0.0121	0.0096	0.0391	0.0058	0.0084
L-10	0.0300	0.0209	0.0077	0.0088	0.0014	0.0120	0.0000	0.0209	0.0043	0.0103
L-11	0.0233	0.0337	0.0111	0.0083	0.0020	0.0036	0.0066	0.0459	0.0176	0.0105
L-12	0.1076	0.1686	0.2418	0.0986	0.1723	0.1926	0.0479	0.3907	0.2456	0.2433
L-13	0.0033	0.0051	0.0026	0.0000	0.0000	0.0161	0.0000	0.0053	0.0020	0.0015
L-14	0.0908	0.3039	0.1120	0.1308	0.1843	0.1337	0.0440	0.1093	0.2384	0.1732
L-15	0.0045	0.0750	0.0037	0.0005	0.0017	0.0000	0.0028	0.0196	0.0050	0.0078
L-16	0.0000	0.0162	0.0000	0.0000	0.0009	0.0000	0.0000	0.0137	0.0020	0.0221
L-17	0.0147	0.0250	0.0028	0.0203	0.0042	0.0442	0.0018	0.0176	0.0205	0.0037
L-18	0.0329	0.0327	0.0718	0.0091	0.0195	0.0241	0.0035	0.0371	0.0372	0.0560
L-19	0.0112	0.0165	0.0018	0.0008	0.0063	0.0139	0.0012	0.0370	0.0120	0.0176
L-20	0.0047	0.0179	0.0008	0.0015	0.0026	0.0227	0.0007	0.0154	0.0206	0.0106
L-21	0.0492	0.0581	0.0134	0.0871	0.0615	0.0593	0.0008	0.0776	0.1316	0.0618
L-22	0.0499	0.0282	0.0064	0.0096	0.0081	0.0092	0.0210	0.0398	0.0199	0.0096
L-23	0.0479	0.0766	0.1634	0.0224	0.0624	0.0485	0.1673	0.0416	0.1767	0.0314
L-24	0.0166	0.0010	0.2641	0.0237	0.0603	0.0494	0.0056	0.0312	0.0323	0.0538
L-25	0.0145	0.0134	0.1116	0.0394	0.0222	0.0587	0.0069	0.0502	0.0544	0.0368
L-26	0.0036	0.0025	0.0969	0.0080	0.0115	0.0042	0.0020	0.0082	0.0384	0.0020

**Table 3:** False positive Dice (FPD) between the manual contour of individual raters and the ground truth for each of the simulated PET lesion. FPD evaluates to 0 for ideal segmentation.

Lesion	Rater									
	R-1	R-2	R-3	R-4	R-5	R-6	R-7	R-8	R-9	R-10
L-1	0.0909	0.2250	0.1078	0.2389	0.1868	0.4868	0.1221	0.1239	0.1593	0.1617
L-2	0.0911	0.1869	0.1670	0.3151	0.1976	0.3405	0.2068	0.1482	0.1288	0.1623
L-3	0.0899	0.2004	0.3353	0.1954	0.2316	0.1838	0.3049	0.1679	0.1278	0.1271
L-4	0.2118	0.2956	0.2402	0.2227	0.2324	0.3136	0.2998	0.2402	0.1983	0.1589
L-5	0.1404	0.3057	0.1499	0.3382	0.2138	0.2324	0.3549	0.1270	0.2017	0.1588
L-6	0.0772	0.0863	0.1754	0.2683	0.2876	0.3095	0.1867	0.0887	0.1298	0.0607
L-7	0.4317	0.4939	0.4987	0.5930	0.5354	0.5238	0.5876	0.3578	0.4741	0.5342
L-8	0.0975	0.0839	0.0800	0.1793	0.0983	0.0389	0.2726	0.0535	0.0332	0.1155
L-9	0.1335	0.2531	0.1088	0.2074	0.2098	0.2286	0.2107	0.1147	0.1710	0.1814
L-10	0.2104	0.1672	0.0799	0.1737	0.2430	0.1360	0.3017	0.1672	0.2044	0.1806
L-11	0.1975	0.2978	0.1900	0.2900	0.3766	0.3963	0.3859	0.1529	0.2044	0.2112
L-12	0.1430	0.1661	0.0964	0.1753	0.1998	0.3709	0.2662	0.0755	0.1930	0.1145
L-13	0.3013	0.1927	0.1778	0.2362	0.2702	0.2453	0.5401	0.1433	0.1745	0.1895
L-14	0.2243	0.1725	0.1112	0.1185	0.1501	0.1840	0.3215	0.1342	0.1051	0.1286
L-15	0.2904	0.2033	0.1527	0.2575	0.2484	0.4055	0.3109	0.1211	0.1197	0.3325
L-16	0.3445	0.2647	0.2868	0.2842	0.3334	0.3932	0.5004	0.1993	0.2628	0.1152
L-17	0.1314	0.1657	0.1405	0.1322	0.1619	0.1068	0.1869	0.1068	0.0686	0.1795
L-18	0.2274	0.2819	0.1948	0.2855	0.2545	0.3361	0.2955	0.1626	0.1136	0.1442
L-19	0.2208	0.1961	0.1900	0.1666	0.1577	0.1603	0.2866	0.0873	0.1013	0.1679
L-20	0.3720	0.4450	0.2546	0.2222	0.2811	0.2346	0.5253	0.1174	0.1186	0.2652
L-21	0.3167	0.4682	0.2316	0.1123	0.1831	0.2170	0.4831	0.0960	0.0688	0.2566
L-22	0.2042	0.4806	0.2502	0.2184	0.2359	0.2910	0.2782	0.1195	0.1326	0.3273
L-23	0.2854	0.3430	0.1743	0.2975	0.2188	0.2344	0.1313	0.2099	0.1026	0.2526
L-24	0.5395	0.6361	0.1831	0.3754	0.3928	0.3317	0.5560	0.3924	0.3001	0.4463
L-25	0.3426	0.5258	0.1767	0.1747	0.2767	0.1775	0.2850	0.1519	0.1337	0.2565
L-26	0.3999	0.4898	0.1835	0.2363	0.2080	0.2902	0.3281	0.1888	0.1621	0.3243

**Table 4:** Global consistency error (GCOERR) between the manual contour of individual raters and the ground truth for each of the simulated PET lesion. GCOERR evaluates to 0 for ideal segmentation.

Lesion	Rater									
	R-1	R-2	R-3	R-4	R-5	R-6	R-7	R-8	R-9	R-10
L-1	0.0004	0.0009	0.0004	0.0009	0.0007	0.0020	0.0005	0.0006	0.0006	0.0006
L-2	0.0006	0.0010	0.0008	0.0015	0.0009	0.0016	0.0010	0.0007	0.0007	0.0008
L-3	0.0003	0.0005	0.0008	0.0004	0.0005	0.0004	0.0007	0.0005	0.0003	0.0003
L-4	0.0003	0.0004	0.0003	0.0003	0.0003	0.0005	0.0004	0.0003	0.0003	0.0002
L-5	0.0003	0.0006	0.0003	0.0007	0.0004	0.0005	0.0007	0.0004	0.0004	0.0003
L-6	0.0003	0.0003	0.0004	0.0006	0.0007	0.0008	0.0004	0.0002	0.0003	0.0002
L-7	0.0004	0.0005	0.0005	0.0006	0.0005	0.0005	0.0006	0.0003	0.0005	0.0005
L-8	0.0013	0.0021	0.0010	0.0015	0.0018	0.0014	0.0021	0.0016	0.0015	0.0012
L-9	0.0005	0.0009	0.0003	0.0006	0.0006	0.0007	0.0006	0.0004	0.0005	0.0005
L-10	0.0006	0.0005	0.0002	0.0005	0.0006	0.0004	0.0008	0.0005	0.0005	0.0005
L-11	0.0009	0.0014	0.0008	0.0013	0.0017	0.0018	0.0017	0.0008	0.0009	0.0009
L-12	0.0008	0.0011	0.0010	0.0009	0.0012	0.0019	0.0011	0.0012	0.0013	0.0010
L-13	0.0012	0.0007	0.0007	0.0009	0.0010	0.0010	0.0022	0.0005	0.0006	0.0007
L-14	0.0016	0.0021	0.0011	0.0012	0.0016	0.0016	0.0019	0.0012	0.0015	0.0014
L-15	0.0010	0.0009	0.0005	0.0008	0.0008	0.0014	0.0010	0.0004	0.0004	0.0011
L-16	0.0006	0.0005	0.0005	0.0005	0.0006	0.0007	0.0010	0.0004	0.0005	0.0002
L-17	0.0012	0.0016	0.0012	0.0013	0.0014	0.0013	0.0016	0.0010	0.0007	0.0015
L-18	0.0012	0.0015	0.0012	0.0014	0.0013	0.0017	0.0014	0.0009	0.0007	0.0009
L-19	0.0028	0.0025	0.0023	0.0020	0.0019	0.0020	0.0035	0.0014	0.0013	0.0022
L-20	0.0025	0.0031	0.0016	0.0014	0.0018	0.0016	0.0036	0.0008	0.0009	0.0018
L-21	0.0014	0.0020	0.0009	0.0007	0.0009	0.0010	0.0019	0.0006	0.0007	0.0012
L-22	0.0014	0.0030	0.0015	0.0013	0.0014	0.0017	0.0017	0.0009	0.0008	0.0019
L-23	0.0018	0.0023	0.0017	0.0017	0.0015	0.0015	0.0015	0.0013	0.0013	0.0015
L-24	0.0017	0.0020	0.0011	0.0011	0.0013	0.0011	0.0017	0.0012	0.0009	0.0014
L-25	0.0035	0.0055	0.0027	0.0020	0.0029	0.0022	0.0028	0.0019	0.0017	0.0028
L-26	0.0022	0.0028	0.0014	0.0013	0.0011	0.0016	0.0018	0.0010	0.0010	0.0017

**Table 5:** Rand index (RNDIND) between the manual contour of individual raters and the ground truth for each of the simulated PET lesion. RNDIND evaluates to 1 for ideal segmentation.

Lesion	Rater									
	R-1	R-2	R-3	R-4	R-5	R-6	R-7	R-8	R-9	R-10
L-1	0.9995	0.9989	0.9995	0.9989	0.9991	0.9974	0.9994	0.9993	0.9992	0.9992
L-2	0.9993	0.9988	0.9991	0.9982	0.9989	0.9980	0.9988	0.9991	0.9992	0.9991
L-3	0.9996	0.9994	0.9989	0.9994	0.9993	0.9994	0.9991	0.9994	0.9996	0.9996
L-4	0.9996	0.9994	0.9995	0.9995	0.9995	0.9993	0.9994	0.9995	0.9996	0.9997
L-5	0.9996	0.9992	0.9996	0.9991	0.9994	0.9994	0.9990	0.9995	0.9994	0.9996
L-6	0.9996	0.9996	0.9995	0.9992	0.9991	0.9990	0.9994	0.9997	0.9996	0.9997
L-7	0.9994	0.9993	0.9993	0.9991	0.9992	0.9992	0.9991	0.9995	0.9993	0.9992
L-8	0.9986	0.9976	0.9989	0.9983	0.9980	0.9984	0.9976	0.9982	0.9983	0.9986
L-9	0.9994	0.9989	0.9996	0.9992	0.9992	0.9991	0.9992	0.9995	0.9994	0.9993
L-10	0.9992	0.9994	0.9997	0.9994	0.9992	0.9995	0.9990	0.9994	0.9993	0.9994
L-11	0.9989	0.9983	0.9990	0.9984	0.9979	0.9978	0.9979	0.9990	0.9989	0.9989
L-12	0.9991	0.9988	0.9988	0.9990	0.9986	0.9978	0.9987	0.9985	0.9985	0.9988
L-13	0.9986	0.9991	0.9992	0.9989	0.9987	0.9988	0.9971	0.9993	0.9992	0.9991
L-14	0.9982	0.9976	0.9988	0.9986	0.9982	0.9982	0.9977	0.9986	0.9982	0.9984
L-15	0.9988	0.9990	0.9994	0.9990	0.9990	0.9983	0.9987	0.9995	0.9995	0.9986
L-16	0.9992	0.9993	0.9993	0.9993	0.9992	0.9990	0.9987	0.9995	0.9994	0.9997
L-17	0.9986	0.9982	0.9986	0.9986	0.9984	0.9986	0.9982	0.9988	0.9992	0.9982
L-18	0.9986	0.9982	0.9986	0.9983	0.9985	0.9979	0.9983	0.9989	0.9992	0.9989
L-19	0.9969	0.9972	0.9974	0.9978	0.9978	0.9977	0.9959	0.9984	0.9985	0.9976
L-20	0.9969	0.9961	0.9980	0.9983	0.9978	0.9981	0.9953	0.9990	0.9990	0.9979
L-21	0.9983	0.9974	0.9989	0.9992	0.9990	0.9988	0.9975	0.9993	0.9992	0.9986
L-22	0.9984	0.9962	0.9983	0.9985	0.9984	0.9979	0.9980	0.9990	0.9990	0.9976
L-23	0.9979	0.9973	0.9981	0.9979	0.9983	0.9982	0.9983	0.9984	0.9985	0.9982
L-24	0.9978	0.9973	0.9987	0.9986	0.9984	0.9987	0.9977	0.9985	0.9989	0.9982
L-25	0.9959	0.9930	0.9971	0.9977	0.9967	0.9975	0.9967	0.9979	0.9981	0.9968
L-26	0.9973	0.9965	0.9984	0.9985	0.9986	0.9981	0.9978	0.9988	0.9988	0.9979

**Table 6:** Adjusted rand index (ADJRIND) between the manual contour of individual raters and the ground truth for each of the simulated PET lesion. ADJRDIND evaluates to 1 for ideal segmentation.

Lesion	Rater									
	R-1	R-2	R-3	R-4	R-5	R-6	R-7	R-8	R-9	R-10
L-1	0.9386	0.8814	0.9412	0.8790	0.9034	0.7550	0.9313	0.9195	0.9156	0.9173
L-2	0.9336	0.8864	0.9134	0.8392	0.8921	0.8276	0.8919	0.9144	0.9213	0.9122
L-3	0.9285	0.8953	0.8309	0.9006	0.8829	0.9065	0.8469	0.8935	0.9337	0.9328
L-4	0.8880	0.8512	0.8776	0.8845	0.8815	0.8336	0.8454	0.8776	0.8986	0.9123
L-5	0.9173	0.8426	0.9214	0.8298	0.8875	0.8797	0.8202	0.8988	0.8912	0.9164
L-6	0.9383	0.9339	0.9103	0.8649	0.8556	0.8431	0.9062	0.9427	0.9282	0.9446
L-7	0.7837	0.7526	0.7501	0.7029	0.7318	0.7376	0.7056	0.8181	0.7625	0.7324
L-8	0.9018	0.8125	0.9229	0.8885	0.8511	0.8778	0.8487	0.8612	0.8663	0.9072
L-9	0.9120	0.8509	0.9388	0.8957	0.8939	0.8790	0.8892	0.9226	0.9111	0.9046
L-10	0.8792	0.9055	0.9559	0.9083	0.8772	0.9256	0.8484	0.9055	0.8951	0.9041
L-11	0.8888	0.8330	0.8987	0.8497	0.8093	0.7986	0.8023	0.8998	0.8882	0.8883
L-12	0.8740	0.8318	0.8301	0.8622	0.8129	0.7168	0.8420	0.7659	0.7796	0.8202
L-13	0.8467	0.9004	0.9092	0.8811	0.8640	0.8684	0.7281	0.9252	0.9111	0.9038
L-14	0.8412	0.7602	0.8875	0.8743	0.8316	0.8399	0.8157	0.8773	0.8270	0.8479
L-15	0.8517	0.8601	0.9213	0.8702	0.8742	0.7961	0.8422	0.9292	0.9373	0.8288
L-16	0.8271	0.8590	0.8561	0.8574	0.8322	0.8027	0.7490	0.8931	0.8671	0.9310
L-17	0.9259	0.9033	0.9273	0.9226	0.9158	0.9234	0.9043	0.9369	0.9548	0.9071
L-18	0.8688	0.8414	0.8656	0.8515	0.8619	0.8185	0.8493	0.8993	0.9240	0.8991
L-19	0.8817	0.8916	0.9022	0.9146	0.9164	0.9112	0.8532	0.9366	0.9422	0.9055
L-20	0.8096	0.7661	0.8709	0.8869	0.8566	0.8700	0.7341	0.9328	0.9296	0.8606
L-21	0.8159	0.7352	0.8767	0.8997	0.8769	0.8610	0.7564	0.9126	0.8992	0.8398
L-22	0.8718	0.7432	0.8704	0.8849	0.8768	0.8484	0.8490	0.9196	0.9230	0.8299
L-23	0.8319	0.7884	0.8298	0.8386	0.8581	0.8572	0.8495	0.8731	0.8592	0.8567
L-24	0.7206	0.6798	0.7755	0.7994	0.7723	0.8085	0.7178	0.7871	0.8330	0.7488
L-25	0.8187	0.7261	0.8538	0.8913	0.8482	0.8801	0.8518	0.8974	0.9045	0.8511
L-26	0.7964	0.7516	0.8586	0.8767	0.8892	0.8514	0.8334	0.9006	0.8989	0.8353

**Table 7:** Normalized mutual information (NMUTINF) between the manual contour of individual raters and the ground truth for each of the simulated PET lesion. NMUTINF evaluates to 1 for ideal segmentation.

Lesion	Rater									
	R-1	R-2	R-3	R-4	R-5	R-6	R-7	R-8	R-9	R-10
L-1	0.9088	0.8942	0.9336	0.9056	0.9142	0.8477	0.9207	0.8902	0.9175	0.9263
L-2	0.8948	0.8636	0.9191	0.8781	0.8911	0.8761	0.9020	0.8998	0.8984	0.9092
L-3	0.8858	0.9105	0.8874	0.9212	0.9129	0.9244	0.8977	0.8697	0.9382	0.9344
L-4	0.9069	0.9033	0.9119	0.9101	0.9139	0.8717	0.8899	0.9119	0.9224	0.9150
L-5	0.9058	0.8853	0.9285	0.8901	0.9050	0.9046	0.8814	0.8479	0.9011	0.9244
L-6	0.8989	0.8957	0.9253	0.9048	0.9016	0.8905	0.9280	0.9205	0.9236	0.9006
L-7	0.8822	0.8703	0.8694	0.8517	0.8625	0.8647	0.8527	0.8874	0.8741	0.8626
L-8	0.8210	0.6482	0.8566	0.8569	0.7201	0.7426	0.8472	0.7186	0.7205	0.8467
L-9	0.8820	0.8427	0.9294	0.9202	0.9170	0.8938	0.9027	0.8916	0.9202	0.9130
L-10	0.8746	0.9001	0.9483	0.9159	0.9091	0.9225	0.8972	0.9001	0.9144	0.9117
L-11	0.8821	0.8380	0.9025	0.8784	0.8673	0.8593	0.8566	0.8661	0.8883	0.8970
L-12	0.8002	0.7296	0.6895	0.7988	0.7142	0.6394	0.8313	0.5827	0.6497	0.6809
L-13	0.8857	0.9126	0.9215	0.9097	0.9005	0.8800	0.8361	0.9277	0.9234	0.9198
L-14	0.7852	0.6027	0.8023	0.7796	0.7145	0.7521	0.8134	0.7962	0.6837	0.7334
L-15	0.8887	0.8174	0.9287	0.9047	0.9048	0.8696	0.8866	0.9149	0.9373	0.8724
L-16	0.8921	0.8839	0.9054	0.9060	0.8925	0.8813	0.8588	0.9049	0.9072	0.9178
L-17	0.9087	0.8808	0.9248	0.9000	0.9146	0.8774	0.9104	0.9140	0.9262	0.9094
L-18	0.8581	0.8422	0.8186	0.8766	0.8691	0.8394	0.8836	0.8730	0.8902	0.8553
L-19	0.8774	0.8776	0.9047	0.9150	0.9082	0.8948	0.8741	0.8915	0.9214	0.8861
L-20	0.8555	0.8135	0.8953	0.9037	0.8842	0.8655	0.8251	0.9160	0.9079	0.8749
L-21	0.8138	0.7610	0.8883	0.8330	0.8382	0.8302	0.8471	0.8500	0.8029	0.8151
L-22	0.8399	0.7908	0.8883	0.8925	0.8896	0.8719	0.8569	0.8828	0.9051	0.8611
L-23	0.8181	0.7643	0.7250	0.8504	0.8202	0.8329	0.7376	0.8498	0.7401	0.8505
L-24	0.8117	0.8201	0.6405	0.8393	0.7841	0.8145	0.8277	0.8240	0.8461	0.7788
L-25	0.8383	0.7893	0.7681	0.8560	0.8462	0.8299	0.8683	0.8503	0.8521	0.8314
L-26	0.8544	0.8346	0.7918	0.8908	0.8936	0.8820	0.8759	0.9048	0.8702	0.8768

**Table 8:** Normalized variation of information (NVARINFO) between the manual contour of individual raters and the ground truth for each of the simulated PET lesion. NVARINFO evaluates to 0 for ideal segmentation.

Lesion	Rater									
	R-1	R-2	R-3	R-4	R-5	R-6	R-7	R-8	R-9	R-10
L-1	0.2352	0.4146	0.2212	0.4161	0.3409	0.8348	0.2558	0.2983	0.3037	0.2943
L-2	0.2545	0.4084	0.3110	0.5512	0.3858	0.5874	0.3824	0.3152	0.2940	0.3192
L-3	0.2614	0.3611	0.5642	0.3405	0.3961	0.3220	0.5097	0.3739	0.2369	0.2415
L-4	0.3786	0.4896	0.4072	0.3880	0.3950	0.5540	0.5128	0.4072	0.3414	0.3038
L-5	0.2945	0.5274	0.2759	0.5643	0.3845	0.4081	0.5988	0.3508	0.3747	0.2920
L-6	0.2300	0.2446	0.3109	0.4531	0.4819	0.5259	0.3202	0.2153	0.2594	0.2084
L-7	0.7065	0.8179	0.8269	1.0081	0.8954	0.8734	0.9974	0.5938	0.7819	0.8932
L-8	0.3583	0.5436	0.2932	0.4104	0.4793	0.3838	0.5385	0.4291	0.4030	0.3474
L-9	0.3187	0.5138	0.2276	0.3564	0.3642	0.4188	0.3850	0.2844	0.3132	0.3355
L-10	0.4203	0.3353	0.1681	0.3221	0.4156	0.2684	0.5065	0.3353	0.3612	0.3360
L-11	0.3973	0.5796	0.3612	0.5182	0.6484	0.6866	0.6757	0.3640	0.3977	0.3943
L-12	0.4306	0.5386	0.5025	0.4708	0.5954	0.8878	0.5452	0.5938	0.6560	0.5325
L-13	0.5226	0.3503	0.3201	0.4069	0.4620	0.4587	0.9344	0.2707	0.3134	0.3362
L-14	0.5505	0.6878	0.3945	0.4302	0.5419	0.5395	0.6420	0.4288	0.5244	0.4955
L-15	0.5041	0.4822	0.2799	0.4401	0.4287	0.6853	0.5336	0.2618	0.2295	0.5812
L-16	0.5699	0.4754	0.4754	0.4713	0.5546	0.6520	0.8424	0.3665	0.4425	0.2489
L-17	0.2859	0.3643	0.2735	0.2988	0.3130	0.2994	0.3483	0.2501	0.1888	0.3410
L-18	0.4654	0.5543	0.4738	0.5149	0.4852	0.6293	0.5184	0.3673	0.2870	0.3678
L-19	0.4365	0.4066	0.3608	0.3184	0.3183	0.3404	0.5217	0.2596	0.2345	0.3613
L-20	0.6586	0.8162	0.4508	0.3994	0.4996	0.4666	0.9238	0.2585	0.2709	0.4925
L-21	0.6324	0.9069	0.4307	0.3558	0.4340	0.4851	0.8302	0.3158	0.3413	0.5529
L-22	0.4607	0.8962	0.4551	0.4103	0.4356	0.5290	0.5320	0.3047	0.2906	0.5907
L-23	0.5890	0.7277	0.5591	0.5650	0.5024	0.5064	0.4944	0.4551	0.4581	0.5074
L-24	0.9634	1.1222	0.6509	0.6809	0.7683	0.6495	0.9723	0.7230	0.5701	0.8521
L-25	0.6427	0.9700	0.5199	0.4084	0.5469	0.4453	0.5277	0.3886	0.3647	0.5404
L-26	0.6985	0.8566	0.4931	0.4343	0.3958	0.5141	0.5716	0.3573	0.3711	0.5653

**Table 9:** Symmetric mean absolute surface distance (SMASD) between the manual contour of individual raters and the ground truth for each of the simulated PET lesion. SMASD evaluates to 0 for ideal segmentation.

Lesion	Rater									
	R-1	R-2	R-3	R-4	R-5	R-6	R-7	R-8	R-9	R-10
L-1	0.1722	0.3391	0.1679	0.3510	0.2794	0.7419	0.1939	0.2259	0.2433	0.2367
L-2	0.1799	0.3150	0.2452	0.4686	0.3057	0.4944	0.2999	0.2371	0.2163	0.2519
L-3	0.1878	0.2867	0.4773	0.2763	0.3249	0.2635	0.4322	0.2568	0.1814	0.1841
L-4	0.2952	0.3805	0.3074	0.2905	0.3014	0.4269	0.3950	0.3074	0.2546	0.2156
L-5	0.2097	0.4282	0.2024	0.4570	0.2922	0.3530	0.4844	0.2079	0.2794	0.2150
L-6	0.1696	0.1753	0.2566	0.3933	0.4136	0.4374	0.2687	0.1613	0.2020	0.1530
L-7	0.4236	0.4958	0.5116	0.6043	0.5451	0.5202	0.6146	0.3493	0.4751	0.5419
L-8	0.2817	1.6295	0.2225	0.3326	1.5369	1.5060	0.4494	1.5668	1.5479	0.2699
L-9	0.2561	0.4131	0.1769	0.3069	0.3103	0.3362	0.3178	0.2208	0.2590	0.2768
L-10	0.3303	0.2607	0.1192	0.2503	0.3392	0.2036	0.4197	0.2607	0.2895	0.2624
L-11	0.2413	0.3649	0.2218	0.3360	0.4285	0.4466	0.4442	0.2104	0.2446	0.2413
L-12	0.2889	0.3965	0.5740	0.3153	0.6297	0.9012	0.3879	1.0810	0.7835	0.6132
L-13	0.5067	0.3149	0.2885	0.3789	0.4358	0.4128	0.9151	0.2342	0.2818	0.3050
L-14	0.4520	0.6091	0.2716	0.2910	0.3893	0.3746	0.4848	0.2928	0.4248	0.3533
L-15	0.4380	0.3889	0.2224	0.3735	0.3640	0.6302	0.4616	0.1978	0.1764	0.5139
L-16	0.4503	0.3652	0.3708	0.3669	0.4346	0.5036	0.6522	0.2725	0.3403	0.1729
L-17	0.2768	0.3590	0.2772	0.2849	0.3224	0.2858	0.3565	0.2384	0.1697	0.3542
L-18	0.3835	0.4230	0.3604	0.4115	0.3835	0.4934	0.4245	0.2753	0.2083	0.2729
L-19	0.4359	0.3856	0.3555	0.3114	0.3024	0.3115	0.5354	0.2245	0.2028	0.3384
L-20	0.5649	0.7653	0.3548	0.3160	0.4059	0.3559	0.7938	0.1968	0.2022	0.3736
L-21	0.5219	0.6538	0.3021	0.2313	0.2964	0.3392	0.6280	0.1991	0.2297	0.3924
L-22	0.3486	0.7244	0.3534	0.3111	0.3351	0.4156	0.3991	0.2085	0.2039	0.4692
L-23	0.4959	0.6506	2.2790	0.4729	0.4857	0.4490	2.2447	0.3663	2.2057	0.4037
L-24	0.6548	0.7737	3.1770	0.4475	0.6342	0.4215	0.6404	0.4841	0.3503	0.6899
L-25	0.5196	0.8030	1.3124	0.3498	0.4726	0.4473	0.4153	0.2978	0.4093	0.4564
L-26	0.6498	0.8193	1.4170	0.4151	0.3193	0.4511	0.5541	0.3058	0.5323	0.5225

**Table 10:** Average Hausdorff distance (AHDST) between the manual contour of individual raters and the ground truth for each of the simulated PET lesion. AHDST evaluates to 0 for ideal segmentation.

Lesion	Rater									
	R-1	R-2	R-3	R-4	R-5	R-6	R-7	R-8	R-9	R-10
L-1	0.0610	0.1137	0.0563	0.1134	0.0908	0.2487	0.0663	0.0789	0.0795	0.0776
L-2	0.0658	0.1203	0.0807	0.1540	0.1035	0.1643	0.1023	0.0834	0.0778	0.0831
L-3	0.0717	0.0999	0.1529	0.0912	0.1072	0.0871	0.1404	0.1026	0.0624	0.0645
L-4	0.1131	0.1379	0.1107	0.1062	0.1078	0.1541	0.1389	0.1107	0.0934	0.0829
L-5	0.0798	0.1456	0.0737	0.1554	0.1043	0.1276	0.1667	0.1005	0.1016	0.0781
L-6	0.0615	0.0657	0.0839	0.1239	0.1313	0.1492	0.0870	0.0562	0.0683	0.0556
L-7	0.1894	0.2199	0.2171	0.2570	0.2278	0.2262	0.2704	0.1599	0.2057	0.2341
L-8	0.1076	0.8521	0.0818	0.1109	0.8472	0.8238	0.1508	0.8447	0.8345	0.0966
L-9	0.0932	0.1523	0.0588	0.0952	0.0984	0.1198	0.1062	0.0768	0.0829	0.0891
L-10	0.1254	0.0934	0.0427	0.0859	0.1119	0.0735	0.1442	0.0934	0.0969	0.0900
L-11	0.1067	0.1668	0.0936	0.1363	0.1702	0.1853	0.1801	0.0982	0.1041	0.1037
L-12	0.1360	0.2045	0.2976	0.1433	0.3155	0.5549	0.1595	0.6671	0.5114	0.3161
L-13	0.1563	0.0952	0.0838	0.1091	0.1240	0.1320	0.3061	0.0701	0.0824	0.0889
L-14	0.2148	0.3588	0.1147	0.1341	0.1812	0.1717	0.1998	0.1278	0.2187	0.1627
L-15	0.1540	0.1481	0.0732	0.1191	0.1149	0.2216	0.1490	0.0681	0.0596	0.1842
L-16	0.1590	0.1365	0.1290	0.1288	0.1505	0.1799	0.2422	0.0997	0.1191	0.0667
L-17	0.0731	0.1001	0.0683	0.0762	0.0789	0.0793	0.0916	0.0613	0.0442	0.0899
L-18	0.1448	0.1593	0.1456	0.1370	0.1283	0.1829	0.1381	0.0986	0.0743	0.1007
L-19	0.1252	0.1159	0.0950	0.0829	0.0826	0.0890	0.1538	0.0633	0.0560	0.0945
L-20	0.2030	0.2892	0.1248	0.1069	0.1379	0.1406	0.3117	0.0647	0.0678	0.1417
L-21	0.2330	0.3136	0.1158	0.1000	0.1229	0.1434	0.2425	0.0895	0.1041	0.1634
L-22	0.1384	0.2965	0.1176	0.1070	0.1134	0.1492	0.1494	0.0792	0.0735	0.1686
L-23	0.2032	0.2812	1.0839	0.1793	0.2022	0.1797	1.0607	0.1408	1.0418	0.1545
L-24	0.3059	0.3436	1.9996	0.2160	0.3565	0.2102	0.2794	0.2283	0.1762	0.3879
L-25	0.1943	0.3386	0.5923	0.1431	0.1822	0.1867	0.1528	0.1109	0.1734	0.1862
L-26	0.2270	0.2867	0.6619	0.1486	0.1074	0.1515	0.2065	0.1069	0.2395	0.1782

**Table 11:** Mahalanobis distance (MDST) between the manual contour of individual raters and the ground truth for each of the simulated PET lesion. MDST evaluates to 0 for ideal segmentation.

Lesion	Rater									
	R-1	R-2	R-3	R-4	R-5	R-6	R-7	R-8	R-9	R-10
L-1	0.0598	0.0521	0.0376	0.0658	0.0602	0.0849	0.0470	0.0404	0.0892	0.0376
L-2	0.0334	0.1115	0.0538	0.0644	0.1130	0.0900	0.0644	0.1102	0.0906	0.0503
L-3	0.0950	0.0945	0.0978	0.0641	0.0793	0.0744	0.0396	0.0954	0.0662	0.0717
L-4	0.1034	0.1082	0.0683	0.0529	0.0671	0.0901	0.0452	0.0683	0.0879	0.0524
L-5	0.0916	0.0835	0.0752	0.0479	0.0774	0.0865	0.0738	0.1185	0.0498	0.0449
L-6	0.0365	0.0548	0.0627	0.0727	0.0648	0.1187	0.0555	0.0474	0.0445	0.0491
L-7	0.1601	0.1122	0.1295	0.1303	0.1110	0.0886	0.2741	0.1203	0.1253	0.1598
L-8	0.0899	0.4550	0.0954	0.0959	0.4393	0.4264	0.0495	0.3895	0.4319	0.0773
L-9	0.0381	0.2129	0.0922	0.0529	0.1009	0.1412	0.1628	0.0460	0.0688	0.0863
L-10	0.1627	0.1362	0.0354	0.1131	0.0832	0.1259	0.0770	0.1362	0.0833	0.1099
L-11	0.0902	0.1422	0.0719	0.1139	0.0891	0.1451	0.1176	0.0943	0.0519	0.0835
L-12	0.1659	0.2097	0.1505	0.1834	0.1727	0.4722	0.2368	0.2946	0.5069	0.2292
L-13	0.1174	0.0478	0.0693	0.0616	0.0639	0.1840	0.2296	0.0588	0.0686	0.0863
L-14	0.2846	0.4791	0.1793	0.2271	0.3122	0.2664	0.2215	0.2013	0.3357	0.2897
L-15	0.0683	0.0807	0.0118	0.0421	0.0450	0.2217	0.0890	0.0504	0.0297	0.1972
L-16	0.0243	0.1228	0.0503	0.0467	0.1202	0.0681	0.1682	0.0614	0.0646	0.1080
L-17	0.0084	0.0243	0.0093	0.0307	0.0525	0.0818	0.0594	0.0288	0.0601	0.0469
L-18	0.1026	0.0978	0.1807	0.0810	0.1173	0.1737	0.0474	0.1263	0.1158	0.1391
L-19	0.0685	0.0669	0.0378	0.0389	0.0477	0.0722	0.1052	0.0351	0.0473	0.0372
L-20	0.1044	0.0871	0.0527	0.0422	0.0571	0.0865	0.2476	0.0530	0.0331	0.1111
L-21	0.1971	0.2168	0.0911	0.1429	0.1491	0.2008	0.1604	0.0892	0.2023	0.1907
L-22	0.0906	0.1030	0.0544	0.0700	0.0311	0.1141	0.0511	0.0358	0.0406	0.1344
L-23	0.1737	0.1605	0.5270	0.1593	0.0841	0.1010	0.5144	0.1045	0.5055	0.1238
L-24	0.3420	0.4058	0.7340	0.3242	0.2065	0.2389	0.2465	0.2569	0.2931	0.3053
L-25	0.1546	0.2824	0.3373	0.1016	0.0618	0.0833	0.1527	0.1625	0.0797	0.0826
L-26	0.2484	0.2549	0.3244	0.2241	0.1662	0.1969	0.3469	0.2139	0.1520	0.2093

**Table 12:** Absolute volumetric difference (AVD) between the manual contour of individual raters and the ground truth for each of the simulated PET lesion. AVD evaluates to 0 for ideal segmentation.

Lesion	Rater									
	R-1	R-2	R-3	R-4	R-5	R-6	R-7	R-8	R-9	R-10
L-1	0.0617	0.2403	0.1038	0.2695	0.1998	0.6434	0.1139	0.0920	0.1633	0.1729
L-2	0.0517	0.1601	0.1764	0.3683	0.1990	0.4081	0.2211	0.1352	0.1069	0.1625
L-3	0.0382	0.2128	0.4007	0.2137	0.2600	0.1994	0.3597	0.1317	0.1317	0.1282
L-4	0.2225	0.3452	0.2681	0.2410	0.2582	0.3466	0.3409	0.2681	0.2154	0.1540
L-5	0.1231	0.3498	0.1542	0.4057	0.2267	0.2536	0.4265	0.0538	0.2060	0.1635
L-6	0.0322	0.0418	0.1884	0.3089	0.3359	0.3621	0.2059	0.0654	0.1239	0.0113
L-7	0.5505	0.6559	0.6645	0.8430	0.7311	0.7096	0.8322	0.4279	0.6215	0.7290
L-8	0.0006	0.1849	0.0076	0.1482	0.0937	0.1517	0.2806	0.1549	0.1804	0.0484
L-9	0.0964	0.2342	0.1007	0.2314	0.2328	0.2428	0.2235	0.0785	0.1800	0.1892
L-10	0.1982	0.1579	0.0749	0.1796	0.2747	0.1321	0.3553	0.1579	0.2224	0.1861
L-11	0.1907	0.3043	0.1964	0.3278	0.4608	0.4884	0.4680	0.1130	0.2061	0.2230
L-12	0.0360	0.0025	0.1355	0.0797	0.0278	0.1956	0.2450	0.2723	0.0512	0.1209
L-13	0.3500	0.2069	0.1921	0.2679	0.3124	0.2588	0.7400	0.1482	0.1887	0.2075
L-14	0.1430	0.1233	0.0008	0.0121	0.0335	0.0515	0.3221	0.0251	0.1250	0.0436
L-15	0.3335	0.1370	0.1610	0.2947	0.2814	0.5086	0.3643	0.1069	0.1216	0.3877
L-16	0.4162	0.2837	0.3348	0.3313	0.3988	0.4895	0.6674	0.2046	0.3000	0.0976
L-17	0.1239	0.1513	0.1478	0.1185	0.1711	0.0646	0.2040	0.0934	0.0492	0.1927
L-18	0.2155	0.2847	0.1310	0.3207	0.2662	0.3696	0.3419	0.1338	0.0793	0.0922
L-19	0.2340	0.1973	0.2077	0.1808	0.1637	0.1580	0.3329	0.0515	0.0934	0.1624
L-20	0.4500	0.5431	0.2906	0.2481	0.3235	0.2369	0.7110	0.1074	0.1029	0.2916
L-21	0.3087	0.5159	0.2449	0.0255	0.1294	0.1712	0.6355	0.0185	0.0609	0.2159
L-22	0.1671	0.5847	0.2777	0.2331	0.2570	0.3280	0.2950	0.0829	0.1194	0.3776
L-23	0.2694	0.3072	0.0109	0.3189	0.1696	0.2049	0.0353	0.1838	0.0714	0.2487
L-24	0.7079	0.9306	0.0778	0.4267	0.3987	0.3286	0.7593	0.4408	0.3091	0.4883
L-25	0.3925	0.6888	0.0673	0.1450	0.2916	0.1263	0.3229	0.1071	0.0825	0.2468
L-26	0.4942	0.6443	0.0905	0.2576	0.2179	0.3337	0.3895	0.1985	0.1319	0.3842

## 2 Experimental design, materials, and methods

A full description of the methods used for the generation of synthetic PET imaging datasets and the associated ground truth data can be found in previous publications [1,2]. Contouring settings and details on quantitative metrics being used were described in the related research article [3]. In short, PET images being used for manual target delineation assessment consisted of 26 synthetic PET datasets created by using the anthropomorphic Zubal thorax phantom [4] in conjunction with the Monte Carlo based Simulation System for Emission Tomography software package (SimSET) [5]. The PET system modeled was a Siemens Biograph scanner featuring a pixelated block BGO detector with ring radius of 42.1 cm. The emission data produced from SimSET for each dataset was re-binned into 128 × 128 sinograms by single-slice re-binning, followed with slice-by-slice reconstruction using an ordered subset expectation maximization (OSEM) algorithm (8 iterations, 4 subsets). Attenuation correction was carried out by aid of the CT data of the Zubal phantom. The resulting image slices were further convolved with a 5 mm full width at half maximum (FWHM) 3D Gaussian filter for noise suppression. Each image dataset included one PET-positive lesion differing in shape, dimension, and uptake heterogeneity with anatomical location either within the lungs or adjacent to the mediastinum or to the chest wall. The participating raters consisted of 10 radiation oncology physicians with extensive clinical experience in contouring PET-imaged lung lesions as part of the radiation therapy (RT) treatment planning process. MIM 6.7.11™ (MIM Software, Cleveland, OH) was employed as the contouring platform and imaging data provided for contouring included the simulated PET along with a co-registered CT of the Zubal phantom. Manual contours by the raters were extracted and evaluated by reference to their respective ground truth data in terms of the aforementioned accuracy metrics. These analyses were carried out either in MIM 6.7.11™ with the utilization of the “compare contours” MIM tool or in MATLAB™ (Version R2019a, MathWorks Inc.) through using proprietary scripts. Worth of note, the presented data is in relation to the supplementary materials associated with our previous report [1] but extends the assessment of manual contouring accuracy significantly with a considerable augmentation in the category of performance measuring metrics. The study was carried out in a double-blind fashion to guard against potential bias, *i.e.*, the raters were not able to view the work from one another and the rater identities were kept anonymous to the investigators.
